# Exosomal MiR-4261 mediates calcium overload in RBCs by downregulating the expression of ATP2B4 in multiple myeloma

**DOI:** 10.3389/fonc.2022.978755

**Published:** 2022-08-26

**Authors:** Sicheng Bian, Xialin Zhang, Leilei Lin, Lili Sun, Zhibo Guo, Jie Pan, Jiangxia Cui, Hanbing Yao, Jing Xu, Zhuanghui Hao, Yuzhu Wang, Liguo Tong, Xingpeng Bu, Desheng Kong, Nianjiao Liu, Yinghua Li

**Affiliations:** ^1^ Key Laboratory of Cell Transplantation of National Health Commission, Heilongjiang Key Laboratory of Blood and Hematopoietic System, Harbin Medical University, Harbin, China; ^2^ Department of Hematology, The First Affiliated Hospital, Harbin Medical University, Harbin, China; ^3^ Department of Hematology, Shanxi Bethune Hospital, Shanxi Academy of Medical Sciences, Tongji Shanxi Hospital, Taiyuan, China; ^4^ Department of Hematology, Affiliated Yantai Yuhuangding Hospital, Qingdao University, Yantai, China; ^5^ Department of Pathology, Stanford University School of Medicine, Palo Alto, CA, United States; ^6^ Department of Hematology, Xi’an International Medical Center Hospital, Xi’an, China; ^7^ Institute of Hematology, The Second Hospital of Shanxi Medical University, Taiyuan, China; ^8^ Central Laboratory, Shanxi Academy of Traditional Chinese Medicine, Taiyuan, China; ^9^ Department of Geriatrics, Shanxi Bethune Hospital, Shanxi Academy of Medical Sciences, Tongji Shanxi Hospital, Taiyuan, China

**Keywords:** ATP2B4, calcium overload, RBC, multiple myeloma, miR-4261

## Abstract

**Background:**

Hypercalcemia induced by multiple myeloma (MM) affects the biological functions of excitable and non-excitable cells. However, red blood cells (RBCs) regulatory effect on calcium in hypercalcemia is still not fully understood.

**Methods:**

A total of 113 patients with MM osteolytic lesions were studied retrospectively. Flow cytometry and atomic absorption spectroscopy were used to detect calcium content. Immunofluorescence and Western blotting were used to investigate protein expression. GEO and miRNA databases were used to screen miRNAs. Exosomal miR-4261 migration was investigated by Transwell assay. Dual-luciferase assays confirmed the targeting relationship between miR-4261 and ATP2B4. An RBC oxidative stress model was constructed, and Omega-Agatoxin IVA was used to study the role of plasma membrane Ca^2+^-ATPase 4 (PMCA4) in RBCs.

**Results:**

The results showed that MM RBCs had calcium overload, and serum calcium levels increased as the number of RBCs decreased. The expression of PMCA4 in MM RBCs was significantly lower than in normal RBCs. The exosomal miR-4261 produced by MM cells could be transferred to RBCs to downregulate the expression of ATP2B4.

**Conclusions:**

Studies have confirmed that RBCs experience calcium overload in MM with osteolytic lesions, which is related to the downregulation of ATP2B4 by MM exosomal miR-4261.

## Introduction

Hypercalcemia is one of the most common complications of MM. When the serum calcium level is higher than 11 mg·dL^-1^ (2.75 mmol·L^-1^), the condition can be defined as hypercalcemia (1). Studies have found that the leading cause of hypercalcemia in MM patients is disrupting the dynamic balance between bone-resorbing osteoclasts and bone-repairing osteoblasts. This process has been proven to be related to abnormalities in osteoclast-activating factors and osteoblast inhibitory factors secreted by clonal malignant plasma cells (1–3). Clinical research data have shown that >80% of patients with *de novo* and relapsed MM have osteolytic bone lesions (1, 4, 5), but only approximately 30% of patients have hypercalcemia (6). Therefore, it is crucial to clarify the factors that regulate calcium ion levels in the blood of MM patients to understand the mechanism underlying pathological calcium regulation in MM.

PMCA is an ATP-driven Ca^2+^ pump (coding gene: ATPase plasma membrane Ca^2+^ transporting 4, ATP2B4) that is ubiquitously expressed in the plasma membrane of human cells. PMCA plays an essential role in the equilibrium of intracellular calcium homeostasis. The family consists of four PMCA isoforms (PMCA1, PMCA2, PMCA3, PMCA4) encoded by four separate genes (ATP2B1, ATP2B2, ATP2B3, ATP2B4), all of which undergo alternative splicing (7). The four subtypes of PMCA have specific sublocalization and distribution in cells (8–11). PMCA4 predominates in RBCs (accounting for more than 80% of PMCAs in RBCs) to maintain the free calcium concentration in RBCs below approximately 40,000 times the plasma free calcium concentration (12, 13). Therefore, the expression and activity of PMCA4 determine calcium fluctuation in RBCs.

MiRNAs are highly conserved short (less than 25 nucleotides long) noncoding RNA molecules that regulate gene expression; miRNAs function posttranscriptionally, silencing gene expression by binding to the 3’-UTRs of target mRNAs to promote their degradation and/or inhibit protein translation (14, 15). MiRNAs account for only 1~3% of the human genome, but they can fine-tune the gene expression of approximately 50% of all protein-coding genes (16, 17). Many studies have found that the expression of miRNAs is associated with the progression of a variety of human cancers (18–21).

Studies have found that a large number of exosomes secreted by MM cells can carry a variety of proteins, lipids, mRNAs and miRNAs (microRNAs) to realize material transfer between cells and affect the biological functions of target cells, including cell proliferation, drug resistance, angiogenesis and osteoclast activity (22–32).

Therefore, we studied RBC calcium overload in MM patients and focused our research on the effect of MM-derived exosomal miRNAs on RBCs. The miRNA array data of this study came from the GEO public platform (https://www.ncbi.nlm.nih.gov/geo/). Through screening and validation of the miRNA array data, we confirmed that miR-4261 regulated the expression of PMCA4 in RBCs. At present, there are only a few studies on miR-4261, and they have mainly been related to the drug resistance of tumor cells such as colorectal cancer, esophageal squamous cell carcinoma, and breast cancer (33–36). However, it is not clear how miR-4261 affects calcium homeostasis in MM RBCs.

This study determined the effect of MM-derived exosomal miR-4261 on the expression of PMCA4 through the detection of clinical samples and *in vitro* experiments. At the same time, the role of PMCA4 in maintaining RBC calcium homeostasis and ROS was studied. The purpose of this study was to prove that RBCs play a vital role in the dynamic balance of free calcium in the blood, which has important pathophysiological significance for understanding MM-induced hypercalcemia.

## Materials and methods

### Patient samples

We reviewed the clinical information of 113 patients newly diagnosed with MM at the Second Hospital of Shanxi Medical University from 2013 to 2021. All MM patients had osteoclastic bone lesions. The sample type used in the experiment was the discarded peripheral blood of newly diagnosed MM patients with osteoclastic bone lesions. This study was performed in line with the principles of the Declaration of Helsinki. Approval was granted by the Ethics Committee of the Second Hospital of Shanxi Medical University (code (2020):YX(076), date of approval: 20200414).

### Cell culture

HEK293T cells were purchased from the Stem Cell Bank of the Chinese Academy of Sciences. Human myeloma RPMI8226 cells and NCI-H929 cells were purchased from Cobioer Biotechnology Co., Ltd. Normal mature RBCs were isolated from peripheral blood by the authors of this study. Cells were cultured with RPMI-1640 medium (Gibco BRL, USA) for suspension cells and high-glucose DMEM (Gibco BRL, USA) for adherent cells supplemented with 10% fetal bovine serum (FBS) (04-001-1 ACS, BI, USA). The MM cell line and RBCs were cocultured in 24 mm Transwell^®^ chambers with 0.4 μm pore polycarbonate membrane inserts preloaded in 6-well culture sterile plates (3412, Corning, USA). The cell culture medium used for cell coculture was serum-free medium (Gibco AIM-V medium CTS, USA). In parallel experiments, serum-free medium was used to culture the myeloma cell line RPMI8226 or H929 cells separately, and the cell culture supernatant was separated and collected at 24 hours. These supernatants were cocultured with normal RBCs. The control group consisted of normal donor RBCs cultured separately in serum-free medium.

### RBC membrane extraction

One milliliter of pure water was added to 500 µL of the RBC pellet, which was obtained by separating peripheral blood from lymphocyte separation fluid. After a waiting period, complete hemolysis was confirmed to have occurred. The suspension was then centrifuged at 15,000 × g for 25 minutes at 4°C, the supernatant was discarded, and the precipitate was retained. The RBC membrane pellet was repeatedly washed with pure water until the supernatant appeared colorless and transparent after centrifugation.

### RNA isolation, reverse transcription and real-time quantitative polymerase chain reaction

(a) Total RNA was extracted from RBCs and WBCs with a total RNA extractor (RNAiso Plus, TaKaRa, Japan). (b) Plasma-free miRNA was extracted using a miRNeasy Serum/Plasma kit (217184, Qiagen, Germany). (c) Exosomal RNA was extracted as follows: Five hundred microliters of Total RNA Extractor was added to 100 µL of exosome solution obtained by ultrafast separation, and 10 µL of RNA-EZ Reagents K RNA-Be-Down (Sangon, Shanghai, China) was added at the same time. The subsequent steps were the same as the abovementioned total RNA extraction steps.

For total RNA reverse transcription, cDNA was generated using Primer-Script RT Master Mix (RR036A, TaKaRa, Japan). For miRNA reverse transcription, cDNA was generated with the Mir-X™ miRNA First-Strand Synthesis Kit (638315, TaKaRa, Japan).

An ABI 7500 Real-Time PCR System was used to detect the reaction system configured by FastStart Universal SYBR^®^ Green Master Mix (ROX) (Roche, Germany). Primer synthesis was performed by Sangon Biotech (**Supplementary Tables 1**, **2**).

### Flow cytometry

The samples were separated into RBCs and WBCs. The RBC labeling antibodies were CD45-PE/Cy7 (IM3548, Beckman Coulter, USA), CD36-PE/Cy5 (Customized from BD Biosciences, USA), CD138-PE (A40316, Beckman Coulter, USA), and Fluo-4 AM (S1060, Beyotime, Shanghai, China), and the WBC labeling antibodies were CD45-PE/Cy 7, CD15-PE/Cy5 (B49217, Beckman Coulter, USA), CD11b-PE (IM2581U, Beckman Coulter, USA), and Fluo-4 AM. A Beckman Coulter FC500 flow cytometer was used to excite the fluorescence and collect the signal.

### Sorting of cells

An appropriate amount of fresh bone marrow cell suspension was vortexed with CD71-FITC (Beckman Coulter, USA) and CD45-PE/Cy7 (Beckman Coulter, USA) and incubated for 30 minutes at room temperature in the dark. Erythrocyte lysis buffer was added to remove mature anucleated erythrocytes, the cells were washed with DPBS and the cell concentration was adjusted to 10^7^~10^8^·mL^-1^. Then, CD71+/CD45- bone marrow nucleated erythrocytes were sorted using FCM.

### Flame atomic absorption spectroscopy

The cells in the samples were counted, and the protein concentration of the cell suspension was determined by the Bradford method. Then, 800 µL 70% HNO_3_ + 200 µL 30% H_2_O_2_ was added to 200 µL cell suspension and digested at 90°C for 3 hours. Five milliliters of pure water were added to the digested sample for dilution. Then, 500 µL of the diluted sample was taken to load the sample, and F-AAS (Z-2000, Hitachi, Tokyo, Japan) was used to detect the total atomic absorbance of calcium.

### Western blot

A 10% SDS-polyacrylamide gel was used for protein electrophoresis, and the proteins were transferred to a 0.45 μm PVDF membrane (Roche, UK) by a wet transfer instrument. Difco™ Skim Milk (232100, BD, USA) was used for nonspecific blocking. FluorChem FC3 was used to collect images of the membrane after ECL incubation. ImageJ software was used for gray value analysis. The antibodies used and dilution ratio were as follows: PMCA4 antibody (NB300-569, NOVUS), 1:1000; Anti-β-Tubulin Mouse Monoclonal Antibody (A01030, Abbkine), 1:5000; Anti-CD138 (WL04081, Wanleibio), 1:500; Anti-CD36 (WL02390, Wanleibio), 1:500; GAPDH Rabbit pAb (AC027, ABclonal), 1:5000; CD9 antibody (sc-13118, Santa Cruz), 1:750; CD63 antibody (sc-5275, Santa Cruz), 1:750; CD81 antibody (sc-166028, Santa Cruz), 1:750; mouse anti-rabbit IgG-HRP (sc-2357, Santa Cruz), 1:5000; and m-IgG κ BP-HRP (sc-516102, Santa Cruz), 1:5000.

### Immunofluorescence staining

The RBC smear was fixed with methanol, permeabilized with saponin (P0095, Beyotime, Shanghai, China) and blocked with QuickBlock™ blocking buffer (P0260, Beyotime, Shanghai, China). The erythrocyte membrane was fluorescently labeled with 10 µM working solution of DiD (D4019, US Everbright, Suzhou, China). A TCS SP8 X confocal laser was used to observe fluorescence at 405 nm and 633 nm excitation. The antibody used and dilution ratio was as follows: PMCA4 antibody (NB300-569, NOVUS), 1:400; and anti-mouse IgG (H+L), CF™ 405S antibody produced in goat (SAB4600023, Sigma–Aldrich, USA), 1:100. ImageJ software was used to measure the fluorescence intensity in pictures of RBCs quantitatively.

### Immunocytochemical staining

PMCA4 protein on the RBC membrane was labeled with PMCA4 antibody (NB300-569, NOVUS) at a dilution ratio of 1:400. The secondary antibody was labeled with a goat anti-mouse IgG H&L/AP antibody (bs-0296G-AP, Bioss) at a dilution ratio of 1:500. Then, the phosphatase substrate BCIP/NBT (C3206, Beyotime) was added to the reaction.

### Bioinformatics analysis

“Myeloma” was used as the search term to obtain the human myeloma cell miRNA expression chip (GEO accession: GSE125364) in the GEO database. “Red blood cells” were used to screen human RBC miRNA expression chips, and 6 sets of chip data were obtained (GEO accession: GSE11060/GSE98830/GSE32035/GSE114990/GSE63703/GSE65706). The miRNAs with 0 expressions in the 6 groups of RBC miRNA chip data (GEO accession: GSE63703/GSE65706) were combined to obtain a collection of miRNAs not expressed in RBCs. The miRNAs expressed in RBCs in these chips were combined (GEO accession: GSE11060/GSE98830/GSE32035/GSE114990/GSE63703/GSE65706) to obtain the RBC expression miRNA data set. In the miRDB and TargetScanHuman_7.2 databases, “ATP2B4” was used as a search tool to obtain miRNA data targeting the ATP2B4 gene. Venn diagram analysis of the above five sets of data was performed to obtain a miRNA data set that also met the following conditions: expression in myeloma cells/lack of expression in RBCs/targeting ATP2B4 (**Supplementary Table 3**).

From the GEO database (https://www.ncbi.nlm.nih.gov/geo/), “red blood cell” was used as the search term to screen the human RBC expression gene chip data (GEO accession: GSE3674). DAVID Bioinformatics Resources 6.8 (https://david.ncifcrf.gov/tools.jsp) was used to enrich and analyze the 8512 RBC-expressed genes to obtain a protein set with calcium binding ability. Simultaneously, the expression levels of four calcium channel proteins (ATP2B1, ATP2B2, ATP2B3, ATP2B4) in the RBC membrane were analyzed. GAPDH was selected as the control.

### Exosome separation and transmission electron microscopy

Five milliliter serum samples were diluted with 30 mL PBS, and then the solution was centrifuged at 2,000 × g for 15 minutes to collect the supernatant. Then, the solution was centrifuged at 10,000 × g for 30 minutes at 4°C to collect the supernatant. Next, the supernatant was filtered with a 0.22 μm syringe filter, transferred for ultracentrifugation (CP70NE, Himac, Japan) in a dedicated ultra isolation tube, and then centrifuged at 120,000 g at 4°C for 90 minutes. The exosome pellet was resuspended in PBS, and the supernatant was analyzed for trace RNA samples in the serum after the exosomes were removed.

Then, 20 µL of the exosome suspension was added dropwise to a 150-mesh carbon-film copper mesh and allowed to stand for 5 minutes. Then, 2% phosphotungstic acid was dropped onto the carbon-film copper mesh and allowed to stand for 1~2 minutes, and the mixture was dried at room temperature. Images were observed under TEM (HT7800, Hitachi, Japan) and collected for analysis. Exosome particle size analysis was performed using Image-Pro Plus 6.0 (Media Cybernetics, Rockville, MD, USA) to measure the exosome diameter (nm) with a 200 nm scale magnified by 40k× as the standard.

### Plasmid construction and dual-luciferase reporter assays

Based on the base sequence that the 3’-UTR of ATP2B4 can recognize with miR-4261, wild-type and mutant ATP2B4 double-stranded DNA fragments and their antisense strands were designed. The reporter gene plasmid psiCHECK-2 (Promega, USA) was connected to the same sticky end. Competent E. coli DH5α was used for transformation, clone colony selection, monoclonal colony amplification, plasmid extraction, and DNA sequencing to identify the extracted plasmid. Using LipoD293™ DNA transfection reagent (SL100668, SignaGen, USA), the wild-type (WT) or mutant (MUT) psiCHECK-2 plasmid vector was cotransfected into HEK293T cells with miRNA mimics or NC (negative control, scrambled sequence). The groups were psiCHECK-2^WT^ + miR-4261 mimics, psiCHECK-2^WT^ + NC, psiCHECK-2^MUT^ + miR-4261 mimics, and psiCHECK-2^MUT^ + NC. Three wells were used for each group. After 48 hours, the Dual-Luciferase Reporter Assay System (E1910, Promega, USA) was used for detection. The measured values of firefly luciferase activity (hLuc fluorescence value) and Renilla luciferase activity (hRluc fluorescence value) were obtained. The synthesis of miRNA mimics and control nucleic acid fragments was commissioned by GenePharma (https://www.genepharma.com/).

### Cellular oxidative stress detection

A reactive oxygen species (ROS) assay kit (S0033S, Beyotime, Shanghai, China) was used to detect RBC ROS, and a hydrogen peroxide (H_2_O_2_) content detection kit (BC3595, Solarbio, Beijing, China) was used to detect the hydrogen peroxide content in RBCs.

### Construction of the RBC oxidative stress model

Tert-butyl hydroperoxide (t-BHP) (458139, Sigma–Aldrich, USA) was diluted to different concentrations with Dulbecco’s phosphate buffered saline (DPBS) solution, and the control group was treated with DPBS solution. Normal RBCs were cultured for 10 minutes, and the following evaluation was performed: pyruvate kinase activity (YX-W-B201, Sinobestbio, Shanghai, China), glucose 6-phosphate dehydrogenase activity (YX-W-A102, Sinobestbio, Shanghai, China), lactate dehydrogenase activity (WLA072, Wanleibio, Shenyang, China), human 2,3-diphosphoglycerate content (YX-041607H, Sinobestbio, Shanghai, China), lactic acid content (A019-2-1, Jiancheng Bioeng, Nanjing, China), malondialdehyde content (WLA048, Wanleibio, Shenyang, China), and ROS level. Each experiment required the Bradford method to determine the RBC protein concentration so that the results could be calibrated.

### Drug suppression experiment

D-Calcium gluconate (C8231, Sigma–Aldrich, USA) was added to the DPBS solution to achieve a working concentration of 1.25 mM (DPBS-C_12_H_22_CaO_14_) (37, 38). Omega-Agatoxin IVA, a Ca^2+^ channel blocker (P/Q type) (ab120210, Abcam, UK), was chosen based on the IC_50_ (2 nM) of its effect on P-type calcium channels. DPBS-C_12_H_22_CaO_14_ was used to adjust the inhibitor working solutions to concentrations of 0, 0.5, 1.0, 2.0, 4.0, and 8.0 nM. Normal RBCs were incubated for 10 minutes, and t-BHP was added to each reaction tube at a final concentration of 0.10 mM. When t-BHP was added at 0, 1, 2, 4, 8, and 10 minutes, Cytation™ 3 was used to detect the fluorescence of Fluo-4 AM-labeled intracellular calcium ions and the level of ROS in RBCs. The Bradford method was used to determine the RBC protein concentration of each reaction system for quantitative calibration (the same method and steps were used for the 250 nM t-BHP treatment test).

### Statistical analysis

Data are presented as the mean ± standard error of the mean (SEM). OriginPro software was used to perform statistical analyses. Nonparametric tests were used for comparisons between two groups, whereas for three or more groups, one-way ANOVA was assumed to be statistically significant for a *P* value < 0.05. Graphs were plotted using OriginPro 2018C software (b9.5.1.195) and SigmaPlot (14.0). The symbols represent significance at * *P* < 0.05, ** *P* < 0.01, *** *P* < 0.001, **** *P* < 0.0001.

## Results

### The effect of the number of RBCs on serum calcium in clinical data

**Figure 1:**
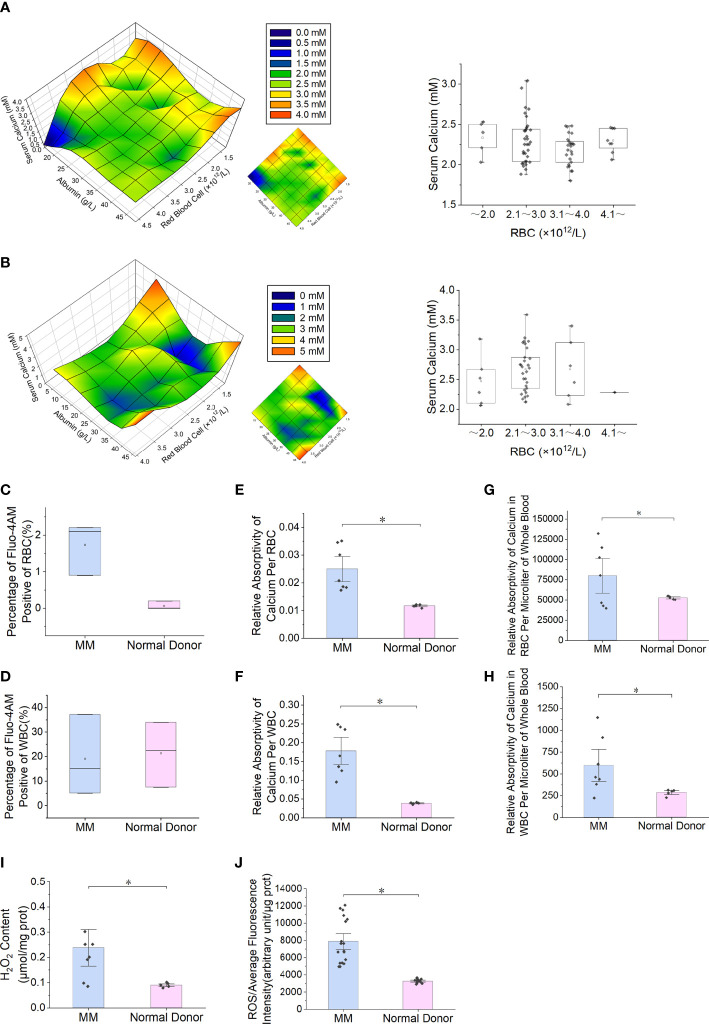
Serum calcium and intracellular calcium levels and RBC oxidative stress in patients with multiple myeloma. **(A)** The patients' serum albumin concentration, serum calcium concentration and the number of RBCs per unit volume of blood are used for three-dimensional composition. In MM patients with normal renal function (number = 71), the serum calcium content increases as the number of RBCs per unit volume decreases. The box chart on the right shows that a high serum calcium level occurs when the number of RBCs is less than 2.0×1012·L-1. When the serum albumin concentration is < 25.0 g·L-1, serum calcium level appears earlier when the number of RBCs is decreased. **(B)** In MM patients with renal insufficiency (number = 42), the analysis results show that the serum calcium level does not increase with the decrease in RBC number. **(C, D)** Flow cytometry (FCM) was used to measure Fluo-4 AM-labeled free calcium ions in RBCs or WBCs. **(E, F)** The total calcium content of single RBCs or WBCs measured by flame atomic absorption spectroscopy (F-AAS) was significantly higher in multiple myeloma than in the normal control group (P = 5.8E-03). **(G, H)** The total calcium content of all RBCs or WBCs per microliter of blood measured by F-ASS was significantly higher in multiple myeloma than in the normal control group (P = 4.4E-02). **(I, J)** Detection of ROS and H2O2 content in RBCs of MM patients and normal controls (PROS = 5.3E-6, PH2O2 = 1.1E-2). (Error bars represent the mean ± SEM, *P < 0.05).

None of the 113 newly diagnosed MM patients with osteolytic lesions had primary kidney disease. Two of the 71 patients with normal renal function (creatinine clearance ≥ 40 mL·min^-1^) had hypercalcemia (serum calcium > 2.75 mmol·L^-1^). The remaining 42 patients had kidney damage (creatinine clearance < 40 mL·min^-1^), which included 13 cases of hypercalcemia (**Table 1**). The relationship between serum albumin concentration, serum calcium concentration and the number of red blood cells per unit volume was observed, and the serum calcium content in MM patients with normal renal function increased significantly with the decrease in the number of RBCs (Pearson correlation coefficient = −0.20) (**Figure 1A**). This phenomenon was not observed for MM patients with renal impairment (**Figure 1B**).

**Table 1:** Clinical data of multiple myeloma with osteolytic bone lesions.

Characteristic		Multiple Myeloma with bone lesions		Total
		Normal GFR	Renal insufficiency

Median age (range) - yr		64 (41-84)	61 (26-85)	64 (26-85)
Male sex - no.		46	26	72
Female sex - no.		25	16	41
Median Weight (range) - kg		64 (40-84.9)	59 (45-80)	63.3 (40-84.9)
Types of Myeloma	IgG-λ	18	2	20
	IgG-κ	13	6	19
	IgA-λ	8	3	11
	IgA-κ	14	5	19
	IgD-λ	2	3	5
	IgD-κ	2	2	4
	λ	5	14	19
	κ	8	6	14
	Non-secretory	1	1	2
D-S system	I	1	0	1
	II	11	2	13
	III	59	40	99
	Renal Failure **^a^**	A	B	/
ISS system	I	13	0	13
	II	36	3	39
	III	22	39	61
Median C-BM-PCs (range) - %		25 (2.4-97.5)	31 (6.5-92.5)	26.75 (2.4-97.5)
Median RBC (range) - ×10^12^·L^-1^		2.91 (1.3-4.62)	2.415 (1.31-4.05)	2.78 (1.3-4.62)
Median Hb (range) - g·L^-1^		95 (47-147)	78 (42-125)	87 (42- 47)
Median ALB (range) - g·L^-1^		30.5 (16.6-48)	37.35 (10.6-46.3)	32.6 (10.6-48)
Median sβ-2MG (range) - mg·L^-1^		4.01 (1.46-38.8)	13.37 (4.17-524.5)	5.8 (1.46-524.5)
Median sCa (range) - mM		2.25 (1.8-3.04)	2.615 (2.06-3.59)	2.31 (1.8-3.59)
Median sCr (range) - μM		83 (42-167)	268 (132.3-1713.2)	100 (42-1713.2)
Median CCr **^b^** (range) - mL·min^-1^		77.32 (43.73-136.03)	23.66 (3.94-37.15)	56.35 (3.94-136.03)

yr, year; kg, kilogram; mM, mmol·L^-1^, μM, μmol·L^-1^; C-BM-PCs, Clonal Bone Marrow Plasma Cells; RBC, Red Blood Cell; Hb, Hemoglobin; ALB, Albumin; sCa, Serum Calcium; sβ-2MG, Serum β-2 Microglobulin; Ca-C-H, Calcium Correction for Hypoalbuminemia; sCr, Serum Creatinine; CCr, Creatinine Clearance Rate; D-S system, Durie-Salmon System; ISS system, International Staging System.

**^a^** Evaluation of renal function injury in D-S system: “A” represents normal renal function (CCr ≥ 40 mL·min^-1^), “B” represents impairment of renal function (CCr < 40 mL·min^-1^).

**^b^** CCr = {[(140-Age) × Weight(kg)]/[0.818 × sCr (μmol·L^-1^)]} × 0.85 (if Female). CCr = mL·minute^-1^, Age = Years, Weight = kg, sCr = μmol·L^-1^.

### RBC calcium overload in MM

The detection of intracellular free calcium by FCM showed that there was no significant difference between RBCs in MM patients and normal subjects (*P* = 0.077), and there was no difference in WBCs (*P* = 1) (**Figures 1C, D**). F-AAS detects the absorbance of calcium in the sample and then calculates the absorbance of calcium in each cell by combining the number of cells with the protein concentration. Comparing the total calcium absorbance in a single cell between the MM patients and controls, the level of WBCs in MM patients was 4.62 times higher than that in normal controls (*P* = 5.8E-03). The RBC level in MM patients was 2.14 times higher than in normal controls (*P* = 5.8E-03) (**Figures 1E, F**). In each microliter of blood, the total calcium absorbance of all MM RBCs was 1.51 times higher than that of control RBCs, and the total calcium absorbance of all MM WBCs was 2.10 times higher than that of normal control WBCs (**Figures 1G, H**).

### RBC ROS is increased in MM

The detection results of ROS (*P* = 5.3E-6) and hydrogen peroxide (*P* = 1.1E-2) in clinical samples showed that the content of MM RBCs was significantly higher than that in normal donors (**Figures 1I, J**).

### Reduced expression of PMCA4 in RBCs from patients with MM

**Figure 2:**
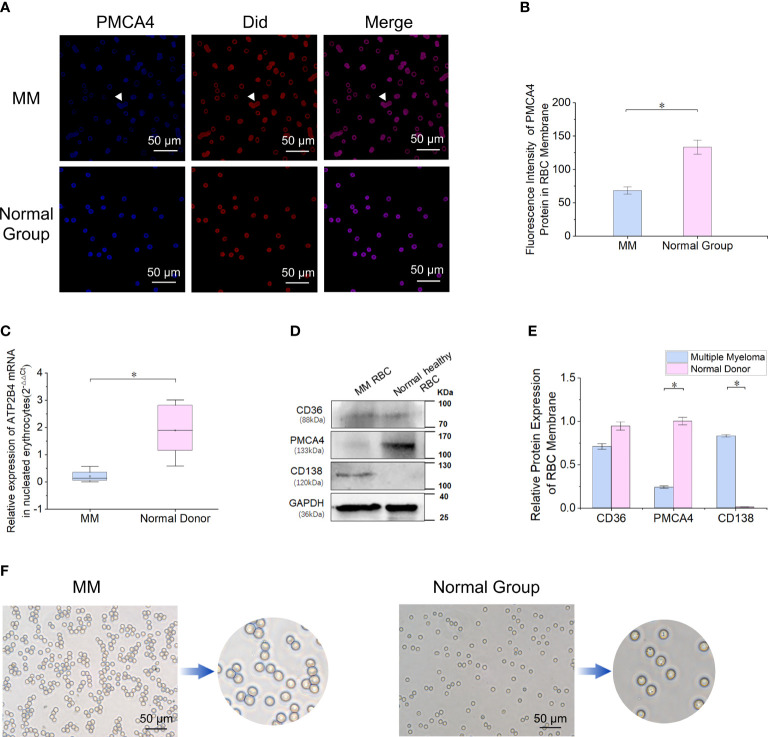
Reduced expression of PMCA4 in RBCs of MM. **(A, B)** Indirect cellular immunofluorescence labeling of the surface of RBCs with PMCA4 (blue), RBC membrane with Did labeling (red), PMCA4 expression in MM RBCs is significantly reduced (*P* = 5.1E-03). The white arrow indicates the stacking of RBCs (rouleaux formation) in MM (magnification, 60×). **(C)** Relative mRNA expression of ATPase plasma membrane Ca^2+^ transport 4 (ATP2B4) in nucleated RBCs of MM bone marrow is significantly lower than that in the normal control group (GAPDH was used as an internal control) (*P* = 5.1E-03). **(D, E)** The expression of PMCA4 in MM RBCs was significantly reduced (*P* = 5.1E-03), and CD138 was expressed. **(F)** After the antibody-labeled RBCs reacted with the substrate BCIP/NBT, the dark blue NBT-formazan deposits in the erythrocyte membrane of MM reacting with PMCA4 protein content were significantly lower than those of the normal control group. (Error bars represent the mean ± SEM, *P < 0.05).

The blue fluorescence intensity of PMCA4 on the surface of MM RBCs was significantly lower than that of normal control RBCs (*P* = 5.1E-03) (**Figures 2A, B**). The relative expression level of ATP2B4 mRNA in nucleated RBCs of MM bone marrow was significantly lower than that in the normal control group (*P* = 5.1E-03) (**Figure 2C**). In addition, the relative expression of PMCA4 protein in the MM RBC membrane was lower than that in the normal control RBC membrane in the separate detection of the pooled samples of the two groups (*P* = 5.1E-03) (**Figures 2D, E**). Immunocytochemical detection of PMCA4 expression also showed that the dark blue NBT-formazan deposits in the RBC membrane of MM were significantly lower than those of the normal control group (**Figure 2F**).

### miR-4261 is highly expressed in RBCs of patients with MM

**Figure 3:**
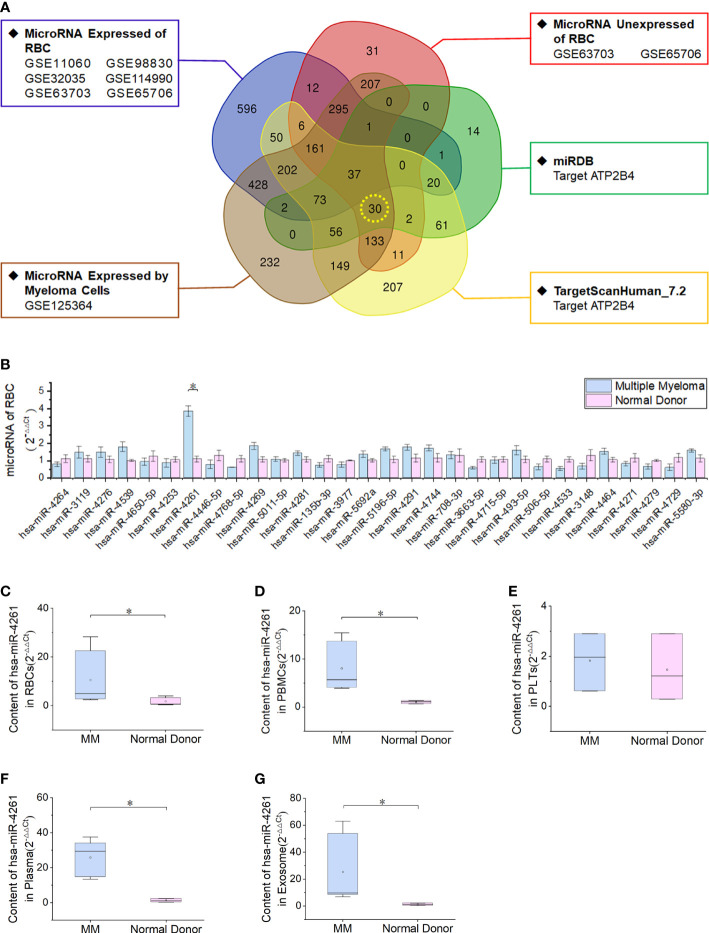
MiRNA-4261 targeting the ATP2B4 gene is highly expressed in MM. **(A)** Screening miRNAs by Venn diagram. The conditions were the following: expression in myeloma cells; lack of expression in normal RBCs; and target gene of ATP2B4. **(B)** qRT–PCR confirms that miR-4261 is highly expressed in MM RBCs (*P* = 9.2E-03). **(C–G)** qRT–PCR was used to measure the content of miR-4261 in various components in blood samples (*P* ^RBC^ = 2.3E-02, *P* ^PBMC^ = 2.3E-02, *P* ^platelet^ = 0.66, *P* ^plasma^ = 3.7E-02, *P* ^exosome^ = 2.8E-02). (Error bars represent the mean ± SEM, *P < 0.05).

By screening RBC miRNA expression data and comparing these data with data in the miRDB and TargetScanHuman_7.2 databases, 30 miRNAs met the following three conditions simultaneously: expression in myeloma cells; lack of expression in normal RBCs; and target gene of ATP2B4 (**Figure 3A**). Enrichment analysis of a human RBC expression chip (GEO accession: GSE3674) revealed that 72 proteins expressed by RBCs possess calcium binding domains (**Supplementary Figure 1**). The expression of ATP2B4 in RBCs was the highest among the four PMCAs (**Supplementary Figure 2**). Clinical samples were used to verify the discovery of the 30 microRNAs screened. The results showed that the relative expression of miR-4261 in MM RBCs was significantly higher than that in normal control RBCs (*P* = 9.2E-03) (**Figure 3B**). We measured the relative content of miR-4261 in various components of peripheral blood again. The results showed that miR-4261 in RBCs (*P* = 2.3E-02), peripheral blood mononuclear cells (PBMCs) (*P* = 2.3E-02), plasma (*P* = 3.7E-02) and serum exosomes (*P* = 2.8E-02) of MM were higher than those of the normal control group, and there was no difference between platelets (*P* = 0.66) (**Figures 3C–G**). The RNA content in the residual supernatant after serum exosome extraction was low and was not detectable.

### Exosomes from MM cells show CD138 expression

**Figure 4:**
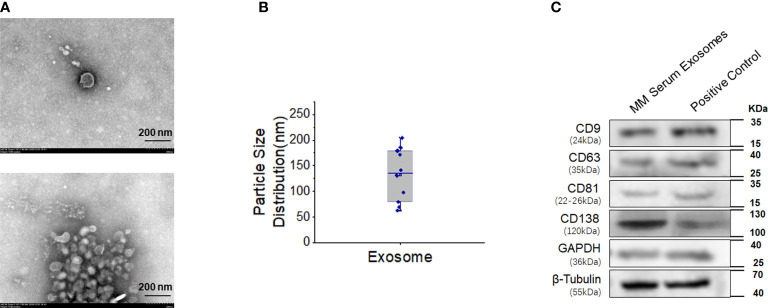
High expression of CD138 in exosomes derived from MM. **(A)** Negative transmission electron microscopy (TEM) staining was used to assess the exosomes extracted from the blood supernatants of MM patients (magnification 40.0k×). **(B)** Diameter analysis of exosomes. **(C)** Western blotting was used to detect the surface marker expression of these exosomes, and the protein extracted from MM bone marrow mononuclear cells were used as the positive control.

The diameter of serum exosomes derived from MM cells was 132.30 ± 16.52 nm (**Figures 4A, B**). Particle size analysis showed that the ratio of particles between 30 and 150 nm was 60%, the ratio of particles between 40 and 100 nm was 40%, and the ratio of particles less than 200 nm was 90%. Western blot analysis showed that the expression of CD138, a marker of clonal plasma cells, was found in the exosomes of MM cells (**Figure 4C**).

### ATP2B4 is a target gene of miR-4261

**Figure 5:**
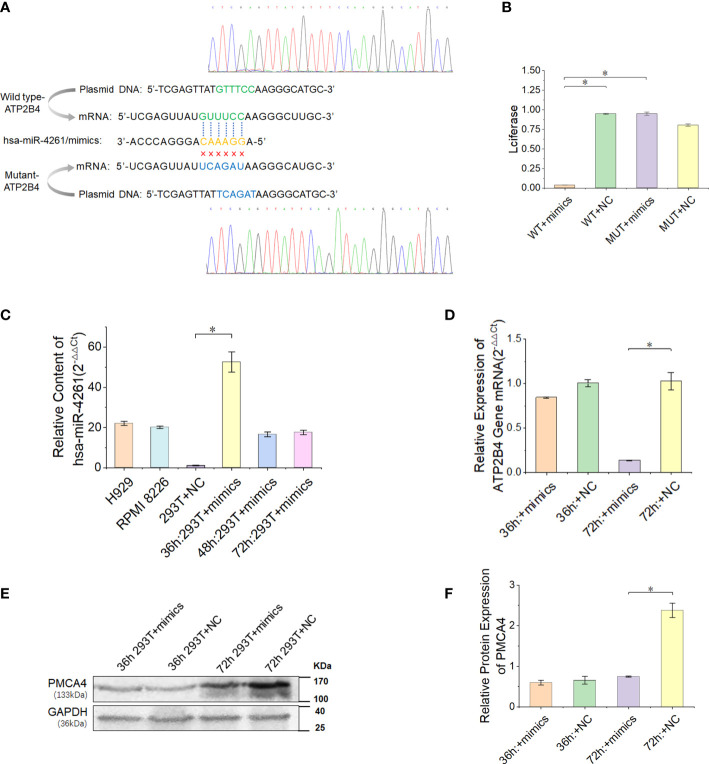
MiR-4261 can target and regulate the ATP2B4 gene and reduce the expression of PMCA4 protein. **(A)** Schematic diagram of the predicted miR-4261 binding site in the 3’-UTR of ATP2B4 mRNA. **(B)** Dual-luciferase reporter assay. The 3’-UTR fragments (wild-type and mutant sequences) of ATP2B4 were cloned into the psiCHECK-2 vector and then cotransfected with miR-4261 mimics or NC. Comparison of relative firefly/Renilla luciferase activity between the wild-type (WT) and negative control (NC) groups (*P* ^WT+mimics vs. WT+NC^ = 1.9E-4, and *P* ^WT+mimics vs. MUT+mimics^ = 1.4E-3). **(C)** The content of miR-4261 in HEK293T cells 36, 48, and 72 hours after transfection of miR-4261 mimics (*P* = 2.7E-2) and the endogenous content of miR-4261 in H929 cells and RPMI8226 cells. **(D)** The mRNA level of ATP2B4 was reduced by miR-4261, and the relative expression decreased most significantly at 72 hours after transfection (GAPDH was used as an internal control) (*P* = 3.6E-2). **(E, F)** MiR-4261 reduced the protein level of ATP2B4 (GAPDH was used as an internal control) (*P* = 4.7E-2). (Error bars represent the mean ± SEM, *P < 0.05).

In the present study, we proved that the expression of ATP2B4 is inhibited by the posttranscriptional regulation of miR-4261 (**Figure 5A**). The experimental results of the double Luciferase Report showed that miR-4261 mimics significantly reduced the fluorescence value of the wild-type plasmid, indicating that miR-4261 can bind to the 3’-UTR of ATP2B4 (**Figure 5B**). The results of gene expression and protein detection showed that miR-4261 significantly inhibited ATP2B4 expression in HEK293T cells, and this effect occurred 72 hours after transfection (**Figures 5C–F**).

### Exosomes secreted by MM cells transfer miR-4261 to RBCs

**Figure 6:**
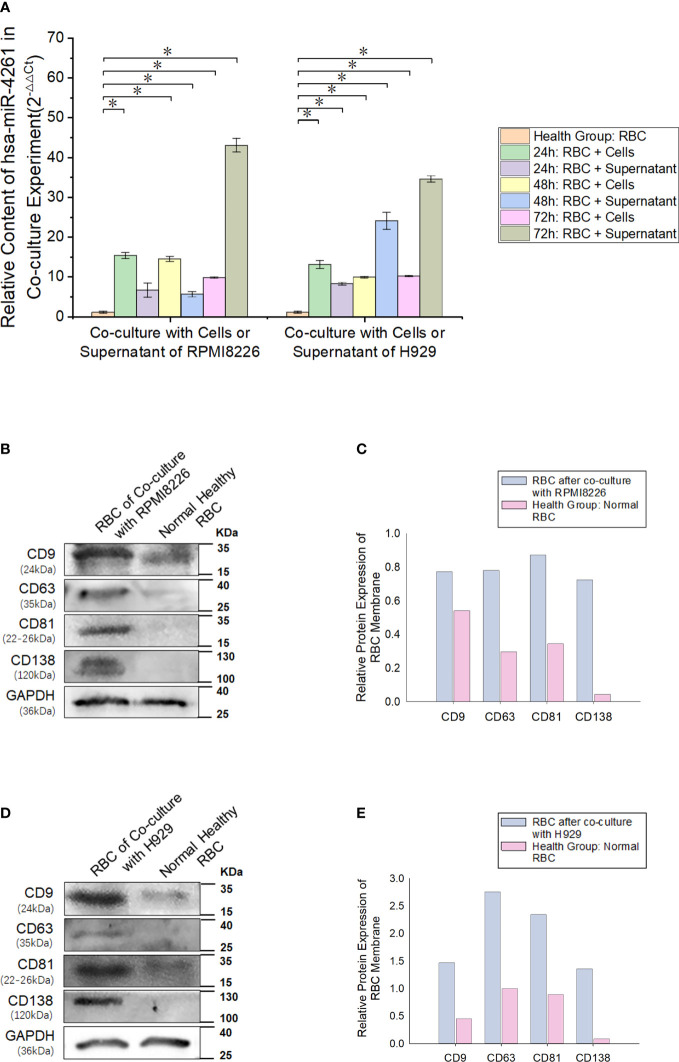
MM cell-derived exosomes can transfer miR-4261 to RBCs. **(A)** Cell coculture experiments show that MM cell lines can deliver miR-4261 to RBCs through the cell culture supernatant (refer to **Supplementary Tables 4** and **5** for *P* values). **(B–E)** The membrane marker CD138 of MM clonal plasma cells can be detected on the surface of normal RBCs after coculture with RPMI8226 or H929 cells, and the marker expression level of exosomes is significantly higher than that of normal RBCs (GAPDH was used as an internal control). (Error bars represent the mean ± SEM, *P < 0.05).

After the MM cell line (RPMI8226 or H929) was cocultured with normal RBCs, the miR-4261 content in RBCs was detected (internal reference: U6). The results showed a significant increase in miR-4261 in normal RBCs after coculture, and the RBCs cultured with the supernatant of the MM cell line showed the same effect (**Figure 6A**). RBCs cocultured with RPMI8226 or H929 cells for 72 hours exhibited higher levels of CD9, CD63, and CD81 than the control group. In addition, CD138 protein was detected in the RBC membrane after coculture (**Figures 6B–E**).

### RBC oxidative stress model

The test results of the RBC oxidative stress model showed that with the increase in the concentration of the t-BHP solution, the RBCs gradually changed from bright red to dark red. When the concentration of t-BHP was higher than 0.75 mM, the erythrocyte sedimentation rate (ESR) gradually increased, accompanied by hemolysis. After performing Wright’s-Giemsa staining on each group of cell samples, it was found that as the concentration of t-BHP increased, RBCs gradually appeared morphologically abnormal, which mainly manifested as abnormal punctate bodies in the cells, and a higher concentration of t-BHP caused the linear body or linear crossover phenomenon to appear. To facilitate the evaluation of the impact of this change on RBCs, we established a scoring system (**Supplementary Table 4**).

Further examination of the damage to RBCs by oxidative stress showed that with the gradual increase in the concentration of t-BHP, the level of ROS in RBCs also gradually increased. MDA, which reflects the lipid peroxidation level of RBC membranes, also steadily increased. However, the content of lactic acid in RBCs gradually decreased. The test results of pyruvate kinase activity and G6-PDH activity also showed that as the concentration of t-BHP increased, the enzyme activity gradually declined. When the concentration of t-BHP was 0.25 mM, LDH activity was reduced by more than half. 2,3-DPG also gradually increased with increasing t-BHP concentration (**Supplementary Figures 3**-**9**).

### Inhibition of PMCA4 can cause RBC calcium overload and increase ROS

RBCs cultured in the calcium-containing reaction system were treated with t-BHP, and the fluorescence signal of Fluo-4 AM-labeled free calcium in RBCs was enhanced. The 0.10 mM t-BHP treatment group peaked at 1 minute, which was maintained for 4 minutes and then rapidly decreased. In the 0.25 mM t-BHP treatment group, the calcium fluorescence signal peak appeared at 2 minutes and was immediately reduced. The calcium fluorescence signal of these two groups of RBCs dropped to the lowest point when t-BHP was incubated for 8 minutes.

**Figure 7:**
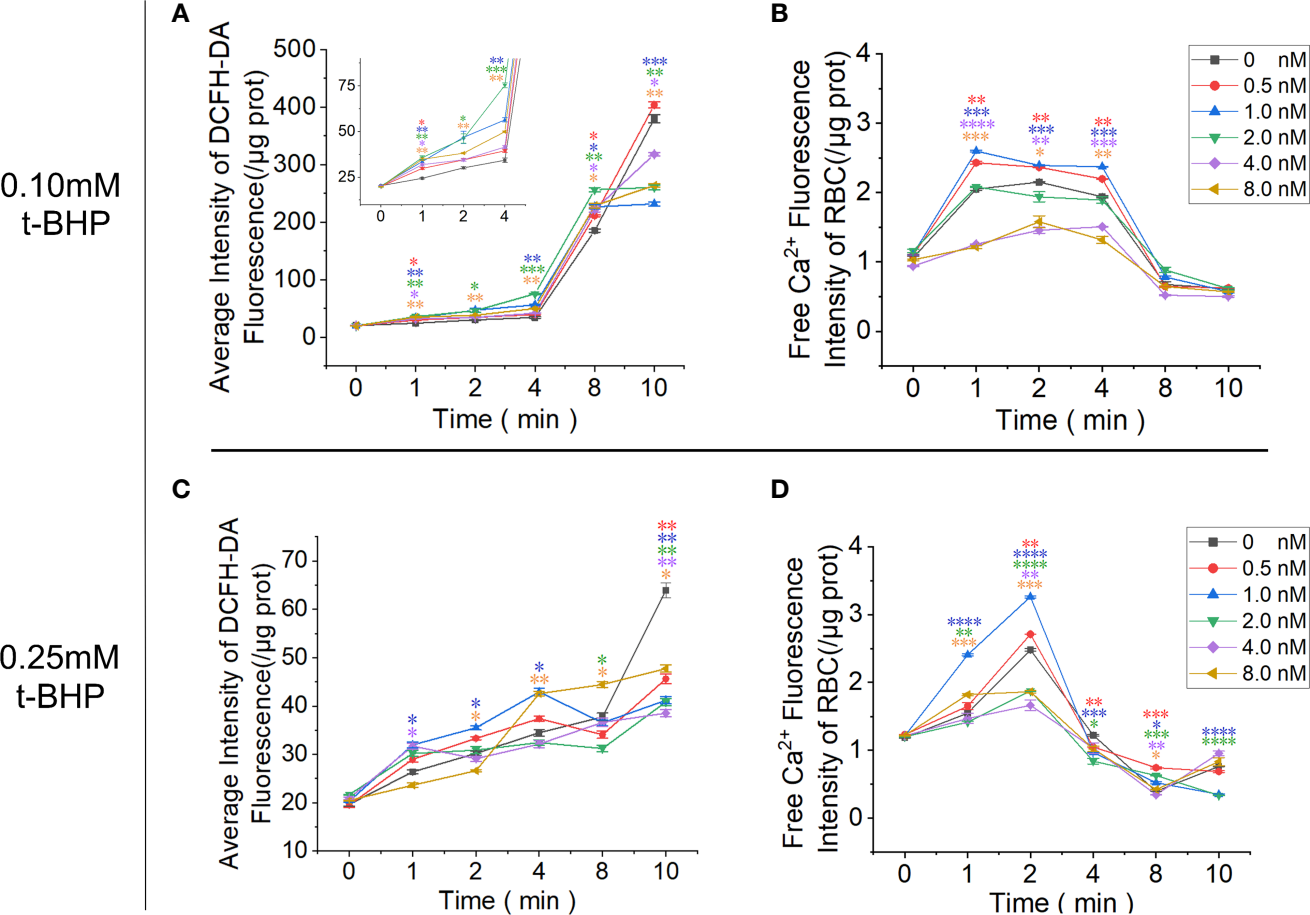
Functional analysis of PMCA4 in maintaining calcium homeostasis in RBCs. RBCs were pretreated with different concentrations of Omega-Agatoxin IVA for 10 minutes and then treated with 0.10 mM or 0.25 mM t-BHP for 0, 1, 2, 4, 8 and 10 minutes prior to the detection of the ROS and free calcium levels in RBCs. **(A)** ROS levels in RBCs after treatment with 0.10 mM t-BHP. **(B)** Free calcium levels in RBCs after treatment with 0.10 mM t-BHP. **(C)** ROS levels in RBCs after treatment with 0.25 mM t-BHP. **(D)** Free calcium levels in RBCs after treatment with 0.25 mM t-BHP. (Error bars represent the mean ± SEM, *P < 0.05, **P < 0.01, ***P < 0.001, ****P < 0.0001 vs. the 0 nM control group. Refer to **Supplementary Tables 5**-**8** for *P* values. One-way ANOVA was used for statistical analysis).

Adding 0.5 nM or 1.0 nM Omega-Agatoxin IVA increased the calcium content of RBCs at each observation time point, but when the inhibitor concentration continued to increase (≥ 2.0 nM), the calcium ion content in RBCs gradually decreased and was even lower than that in the control group. The increase in ROS production in RBCs in the 0.1 mM t-BHP treatment group could be divided into two stages: the increase in ROS production was relatively gradual at 0~4 minutes, and the ROS production in RBCs increased rapidly at 4~8 minutes. The inhibitor Omega-Agatoxin IVA increased the ROS level of RBCs at each time point. However, the ROS in the RBCs increased slowly in the 0.25 mM t-BHP treatment group (**Figure 7**; **Supplementary Table 5**-**8**).

## Discussion

Serum calcium levels in the human body consist of 3 parts: (a) nondiffusible (protein-bound) calcium, which, depending on pH and temperature, represents approximately 46% of the total concentration; (b) diffusible nonionized calcium (i.e., complexes and chelates) comprising approximately 6.5% of the total concentration; and (c) ionized calcium, which accounts for approximately 47.5% of the total concentration (39, 40). Due to the rich content of plasma albumin and a calcium/zinc-binding site on Asp-273, it can bind to serum calcium in a nonspecific way to modulate the concentration of free calcium (41, 42).

In this study, peripheral blood samples of MM with osteolytic lesions ensured that the experimental samples were the source of the pathological increase in calcium ions. Analyses of clinical data of MM patients with normal renal function indicated that the serum calcium content gradually increased with the decrease in the number of RBCs. This phenomenon is not affected by changes in albumin concentration. Therefore, RBCs and albumin may form an “RBC/protein” calcium buffer system to jointly maintain the serum calcium concentration’s stability. We found that the total calcium content in the cells was higher in single RBCs or WBCs from MM patients than in those from the normal control group. It was further found that the total calcium content of all RBCs or WBCs in a single unit volume of blood in MM samples was also significantly higher than that in the normal control. Because the number of RBCs per unit volume of peripheral blood is 1000 times that of WBCs, the calcium retention capacity of RBCs is 100 times greater than that of WBCs. Therefore, the RBCs in MM patients are overloaded with calcium.

The efflux channel that maintains the level of calcium ions in RBCs mainly depends on the PMCA4 protein in the cell membrane (43). We found that the protein expression of PMCA4 on MM RBCs was significantly reduced, and the expression of ATP2B4 was also decreased in the precursor RBCs in the bone marrow. *In vitro* experiments also confirmed our hypothesis that CD138+ exosomes derived from MM cells can transfer miR-4261 to target cells and recognize the 3’-UTR of ATP2B4. Hence, myeloma-derived exosomal miR-4261 can be delivered to RBCs, and miR-4261 can downregulate the expression of ATP2B4 in target cells, leading to a reduction in PMCA4 protein expression. As a part of the microenvironment of MM cells, RBCs become one of the target cells of exosomes secreted by MM cells (27, 32).

When the expression of PMCA4 in the main efflux calcium channel of RBCs is abnormal, it leads to the retention of calcium in the cytoplasm. When free calcium ions abnormally accumulate in the cell cytoplasm and cannot be removed quickly, they become toxic to the cell (44). The most common consequence is the pathological production and accumulation of ROS in the cell. These increased ROS levels can also lead to cellular calcium overload (45). Therefore, abnormal fluctuations in intracellular calcium ion and intracellular ROS levels often result from cross-talk (46). To prove the interaction between the calcium overload caused by low PMCA4 expression in RBCs and high ROS, we designed an RBC oxidative stress model and combined it with PMCA4 channel inhibitors in this study.

In this experiment, the P/Q-type calcium channel protein inhibitor Omega-Agatoxin IVA, a Ca^2+^ channel blocker, was used to study the function of PMCA4 in normal mature RBCs (47). The reaction system uses D-calcium gluconate to simulate the plasma-free calcium ion concentration in the normal controls to prevent interference from the chloride ions introduced by calcium chloride (48). The results showed that the RBCs of the control group without calcium channel inhibitors increased in intracellular free calcium after stimulation with t-BHP, which manifested as an initial increase in intracellular calcium followed by a decrease as oxidative stress stimulation continued. At the same time, under the condition of 0.10 mM t-BHP, there was an obvious plateau in the increase in calcium in RBCs. This finding indicates that the optimal inhibitory concentration of Omega-Agatoxin IVA inhibitor on RBC PMCA4 is 0.5 nM~1.0 nM. Moreover, unlike the 0.1 mM t-BHP treatment group, the free calcium ions in the RBCs of the 0.25 mM t-BHP treatment group did not exhibit a plateau during free calcium ion overload, and the calcium fluorescence signal decreased in advance. We speculate that this phenomenon may be due to Omega-Agatoxin IVA weakening the ability of RBCs to resist oxidative stress, which leads to crosstalk between calcium overload and ROS, aggravates cell damage and the inactivation of proteins and biologically active enzymes and causes the free calcium ion fluorescent tracer Fluo-4 AM not to be hydrolyzed by esterase. In addition, damage from ROS to the phospholipid component of the RBC membrane can also increase the permeability of the cell membrane and cause cell inactivation (49). In the 0.10 mM t-BHP treatment group, the ROS level in RBCs increased rapidly after 4 minutes and was greater than that of the control group without inhibitors. In the 0.25 mM t-BHP treatment group, ROS increased slightly over time, which may be related to the calcium channel inhibitor increasing the intensity of damage to cell membranes by hydrogen peroxide, thereby weakening the ability of RBCs to tolerate the accumulation of ROS.

**Figure 8:**
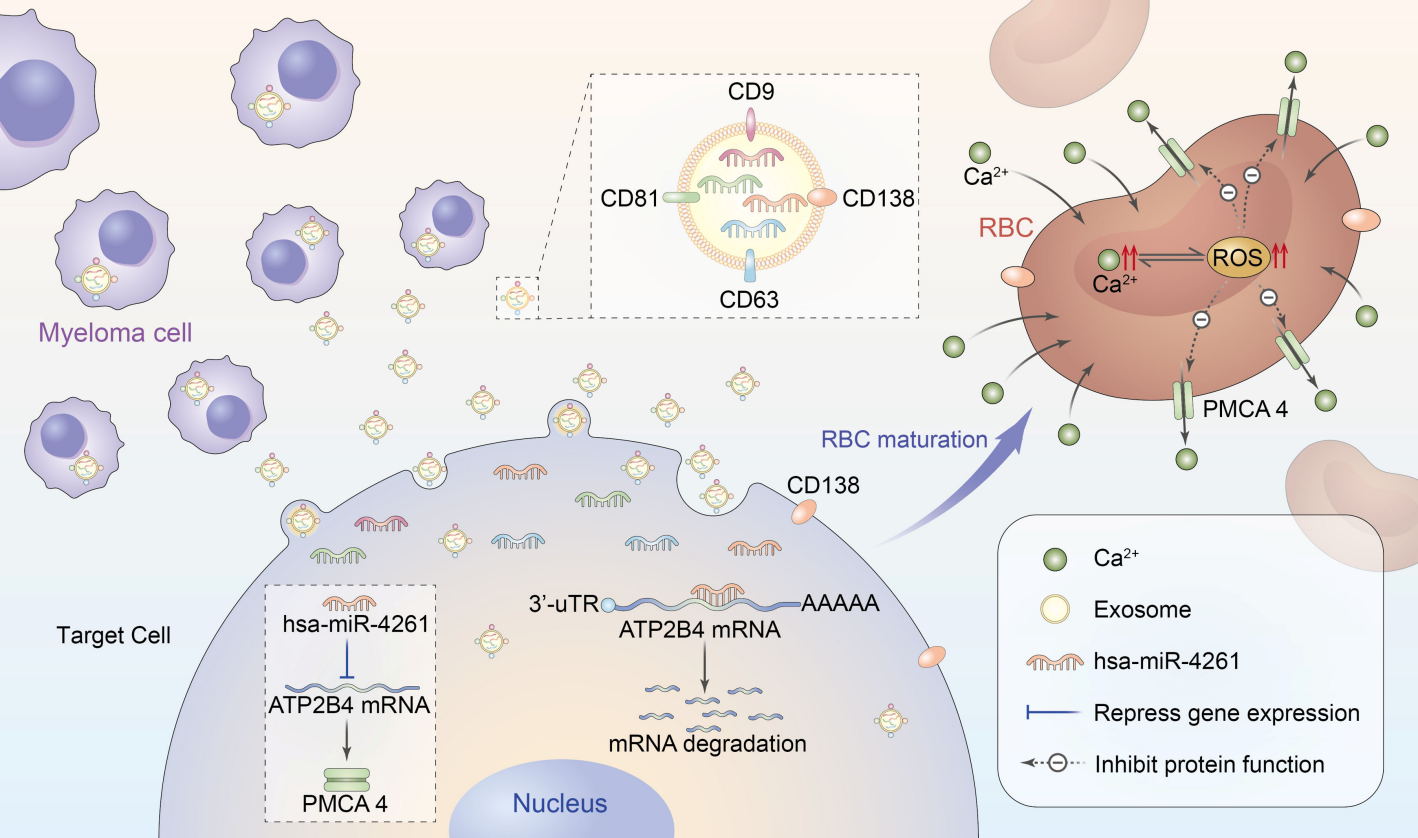
Schematic diagram of RBC calcium overload mediated by MM exosomal miR-4261. Exosomes secreted by MM cells transfer miR-4261 to erythroid precursor cells, resulting in RBC calcium overload by downregulating the expression of ATP2B4. Calcium overload can cause crosstalk that leads to an increase in intracellular ROS levels, forming a vicious cycle that causes further damage to the activity of PMCA4 in RBCs.

In summary, MM cells can deliver miR-4261 to target cells through exosomes, and downregulation of ATP2B4 expression leads to a decrease in PMCA4 content, which inhibits the function of RBCs to excrete calcium and ultimately leads to calcium overload in RBCs (**Figure 8**). Calcium overload can cause crosstalk that increases intracellular ROS levels, forming a vicious cycle that causes further damage to RBCs. Therefore, although MM is a malignant clonal disease of plasma cells, we believe that the RBCs of MM are pathological. However, from another perspective, RBC calcium overload in patients with myeloma alleviates the pathological increase in serum free calcium to a certain extent and reduces the damage of free calcium to important calcium-sensitive cells, tissues and organs. Hence, RBCs can also be considered large free calcium reserve cells in the human body.

## Data availability statement

The original contributions presented in the study are included in the article/**Supplementary Material**. Further inquiries can be directed to the corresponding author.

## Ethics statement

The studies involving human participants were reviewed and approved by the Ethics Committee of the second Hospital of Shanxi Medical University (code: (2020)YX(076), date of approval: 20200414). The patients/participants provided their written informed consent to participate in this study.

## Author contributions

SB, YL Conception and design, data analysis and interpretation, manuscript writing; XZ, LL, NL Data interpretation; JC, XB collection and assembly of data; JP, JX Bioinformatics analysis; LS, ZG, YW Provision of study material or patients; ZH, DK Electron microscope operation; HY, LT Operation of confocal and atomic absorption spectrometry; conception and design, financial support, data analysis and interpretation, final approval of the manuscript. All authors read and approved the final manuscript.

## Funding

This research was funded by the Basic Research Project of Shanxi Province (No. 20210302124037) and Shanxi Bethune Talent Foundation Project (No. 2021RC038).

## Acknowledgments

The authors thank Professor Dongmei Wu (Department of Pharmacology from Shanxi Medical University) for the help in drug design.

## Conflict of interest

The authors declare that the research was conducted in the absence of any commercial or financial relationships that could be construed as a potential conflict of interest.

## Publisher’s note

All claims expressed in this article are solely those of the authors and do not necessarily represent those of their affiliated organizations, or those of the publisher, the editors and the reviewers. Any product that may be evaluated in this article, or claim that may be made by its manufacturer, is not guaranteed or endorsed by the publisher.
