# Supplementary material for: Exosomal MiR-4261 mediates calcium overload in RBCs by downregulating the expression of ATP2B4 in multiple myeloma
Source: Front Oncol. 2022 Aug 26;12:978755. doi: 10.3389/fonc.2022.978755 (PMC9458875; doi:10.3389/fonc.2022.978755)
Supplement: Supplementary file 1 [file DataSheet_1.docx]

Supplementary Material

## Supplementary Figures


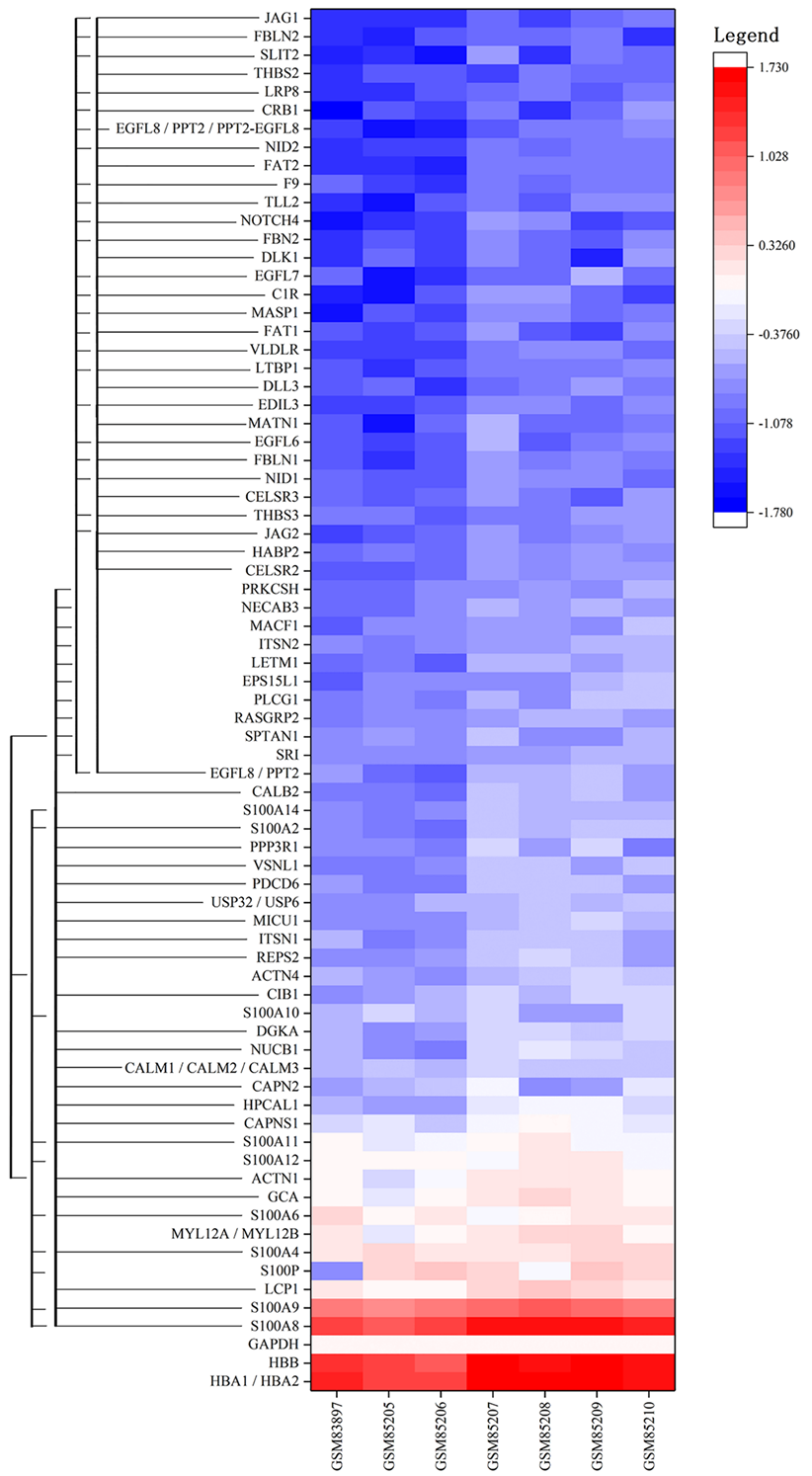


**Supplementary Figure 1.** Seventy-two proteins expressed by RBCs possess calcium-binding domains (GSE3674).


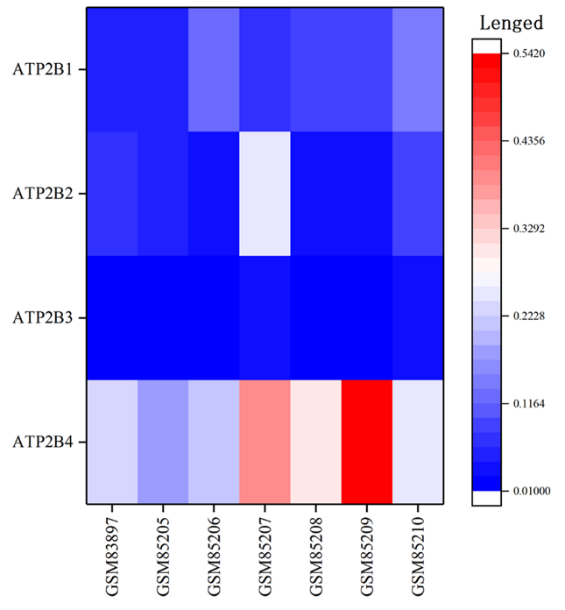


**Supplementary Figure 2.** The relative expression levels of four PMCAs in RBCs (GSE3674).


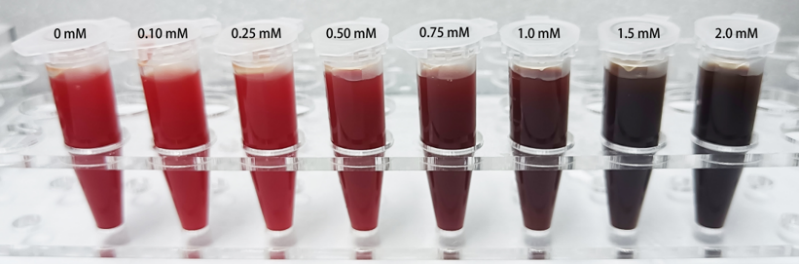


**Supplementary Figure 3.** Color change and RBC membrane change of RBC suspension after treatment with different concentrations of t-BHP.


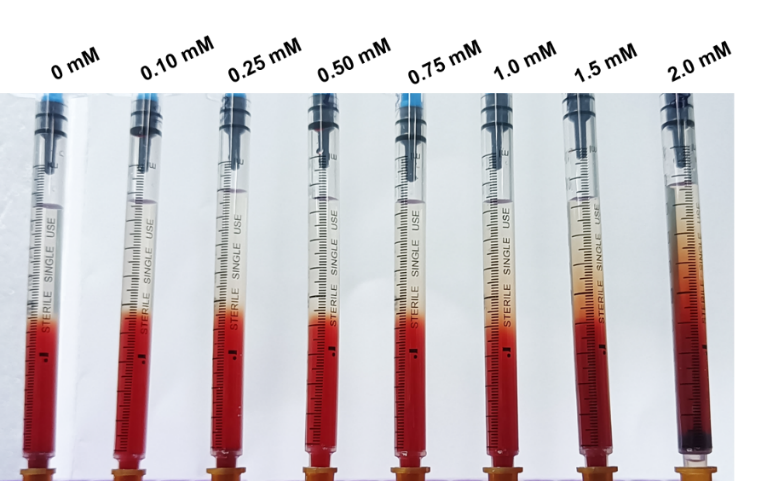


**Supplementary Figure 4.** ESR and hemolysis of RBCs after treatment with different concentrations of t-BHP.


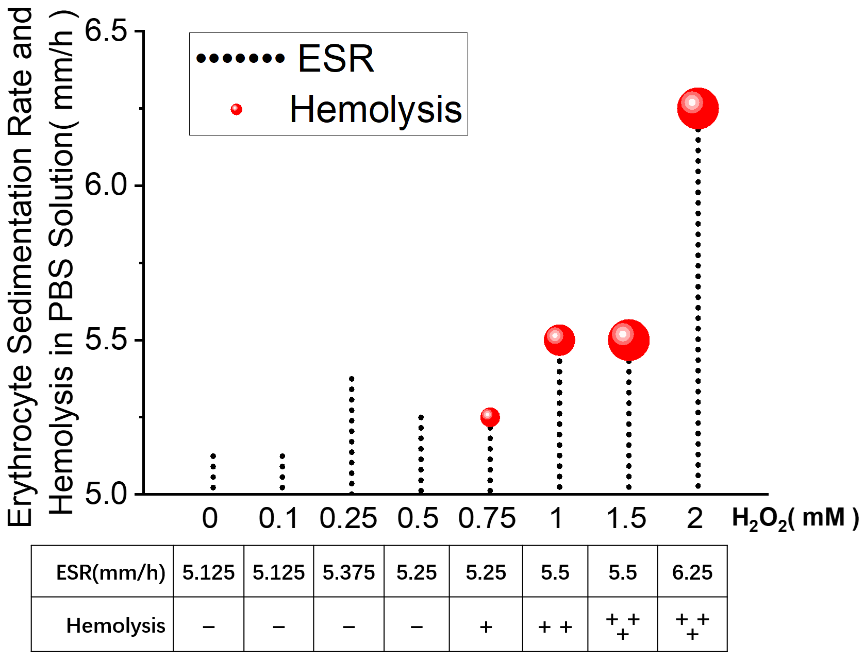


**Supplementary Figure 5.** ESR and hemolysis of RBCs after treatment with different concentrations of t-BHP.


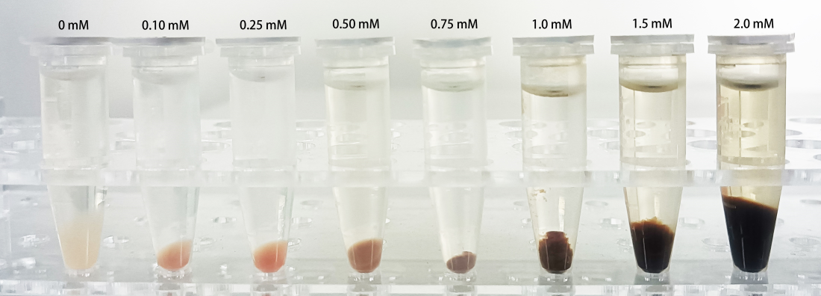


**Supplementary Figure 6.** Color change and RBC membrane change of RBC suspension after treatment with different concentrations of t-BHP.


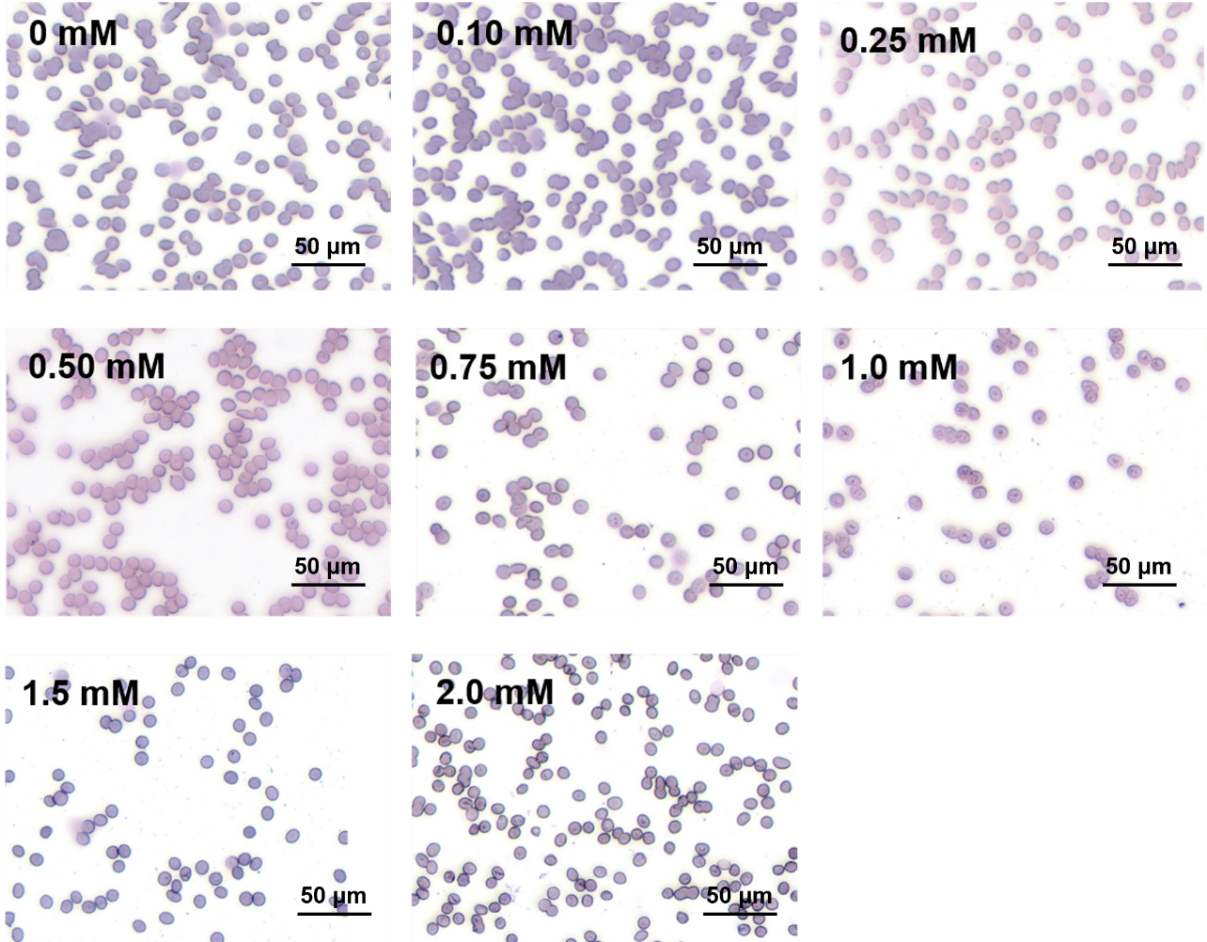


**Supplementary Figure 7.** Morphological changes of RBCs treated with t-BHP. Normal RBCs were treated with different concentrations of t-BHP for 10 minutes, stained with Wright’s-Giemsa staining and observed under a light microscope (magnification, 40×).


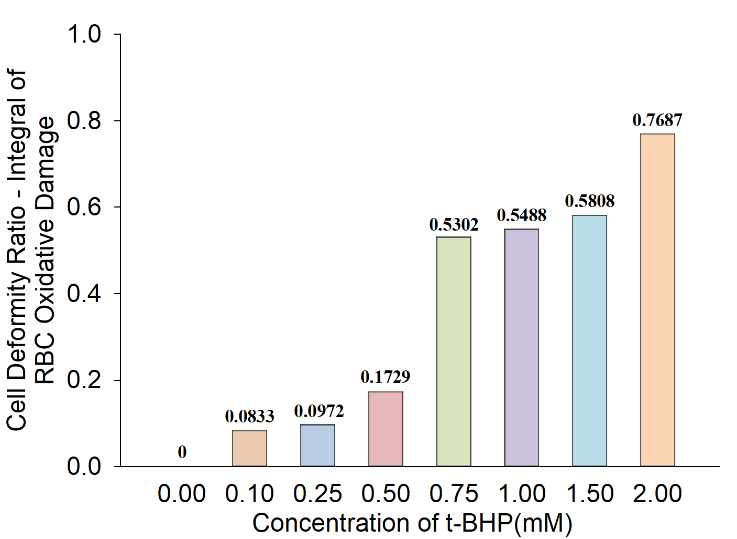


**Supplementary Figure 8.** The RBCs’ oxidative damage score after incubation with different concentrations of t-BHP.


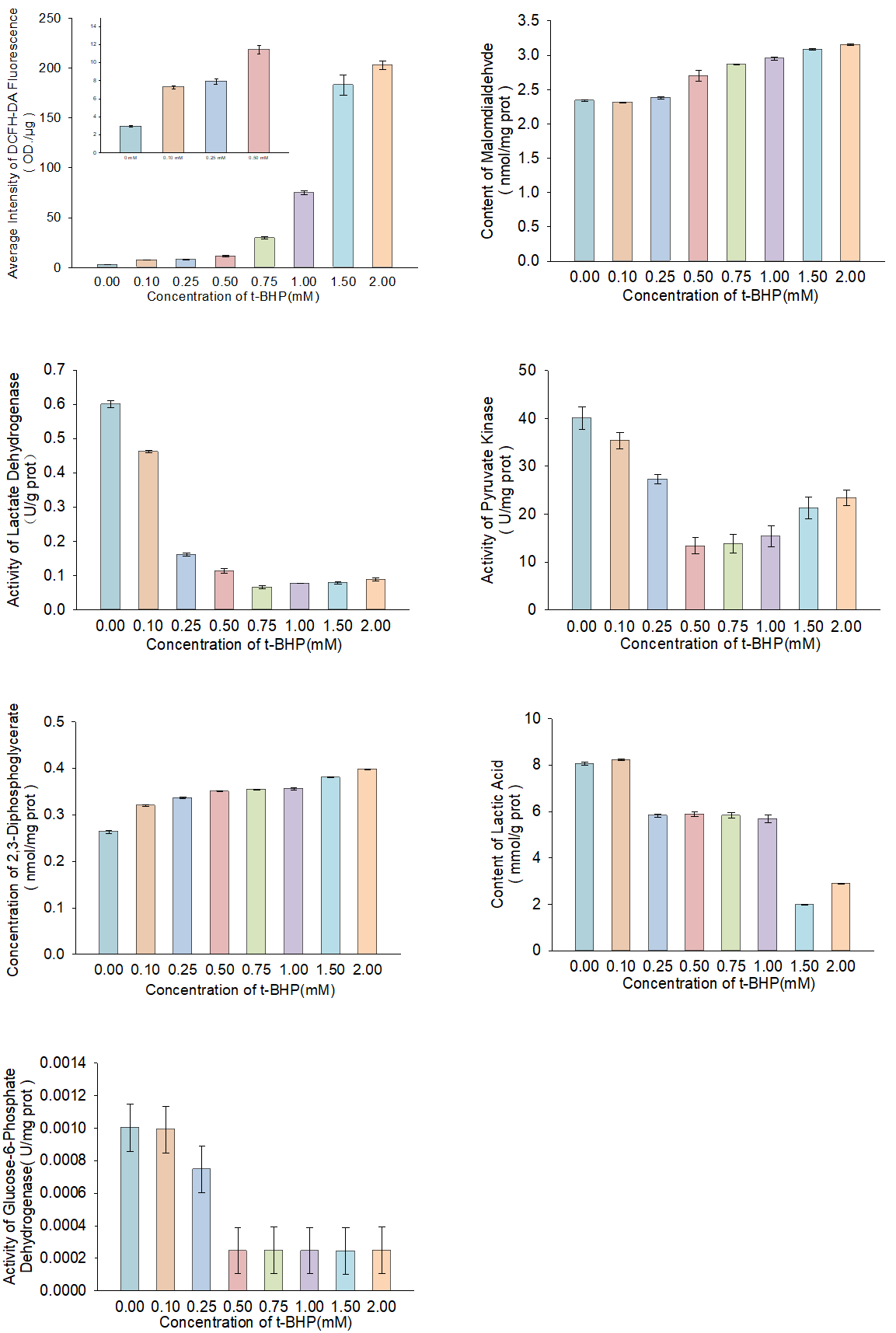


**Supplementary Figure 9.** Test the content or activity of related substances in the RBCs oxidative stress model treated with t-BHP. ROS level, MDA content, lactic acid content, LDH activity, G6-PDH activity, PK activity, and 2,3-DPG content. (prot: protein)(Error bars represent the mean ± SEM.)

## Supplementary Tables

**Supplementary Table 1.** MicroRNA primers for qRT-PCR.

| **miRNAs Name** | **Primer Sequence (5’ to 3’)** |
| --- | --- |
| hsa-miR-4264 Forward | ACTCAGTCATGGTCATT |
| hsa-miR-3119 Forward | TGGCTTTTAACTTTGATGGC |
| hsa-miR-4276 Forward | CTCAGTGACTCATGTGC |
| hsa-miR-4539 Forward | GCTGAACTGGGCTGAGCTGGGC |
| hsa-miR-4650-5p Forward | TCAGGCCTCTTTCTACCTT |
| hsa-miR-4253 Forward | AGGGCATGTCCAGGGGGT |
| hsa-miR-4261 Forward | AGGAAACAGGGACCCA |
| hsa-miR-4446-5p Forward | ATTTCCCTGCCATTCCCTTGGC |
| hsa-miR-4768-5p Forward | ATTCTCTCTGGATCCCATGGAT |
| hsa-miR-4269 Forward | GCAGGCACAGACAGCCCTGGC |
| hsa-miR-5011-5p Forward | TATATATACAGCCATGCACTC |
| hsa-miR-4281 Forward | GGGTCCCGGGGAGGGGGG |
| hsa-miR-135b-3p Forward | ATGTAGGGCTAAAAGCCATGGG |
| hsa-miR-3977 Forward | GTGCTTCATCGTAATTAACCTTA |
| hsa-miR-5692A Forward | CAAATAATACCACAGTGGGTGT |
| hsa-miR-5196-5p Forward | AGGGAAGGGGACGAGGGTTGGG |
| hsa-miR-4291 Forward | TTCAGCAGGAACAGCT |
| hsa-miR-4744 Forward | TCTAAAGACTAGACTTCGCTATG |
| hsa-miR-708-3p Forward | CAACTAGACTGTGAGCTTCTAG |
| hsa-miR-3663-5p Forward | GCTGGTCTGCGTGGTGCTCGG |
| hsa-miR-4715-5p Forward | AAGTTGGCTGCAGTTAAGGTGG |
| hsa-miR-493-5p Forward | TTGTACATGGTAGGCTTTCATT |
| hsa-miR-506-5p Forward | TATTCAGGAAGGTGTTACTTAA |
| hsa-miR-4533 Forward | TGGAAGGAGGTTGCCGGACGCT |
| hsa-miR-3148 Forward | TGGAAAAAACTGGTGTGTGCTT |
| hsa-miR-4464 Forward | AAGGTTTGGATAGATGCAATA |
| hsa-miR-4271 Forward | GGGGGAAGAAAAGGTGGGG |
| hsa-miR-4279 Forward | CTCTCCTCCCGGCTTC |
| hsa-miR-4729 Forward | TCATTTATCTGTTGGGAAGCTA |
| hsa-miR-5580-3p Forward | CACATATGAAGTGAGCCAGCAC |
| mRQ 3’ Primer | provided by Mir-X^TM^ miRNA First-Strand Synthesis Kit (Code No 638315, TaKaRa) |
| U6-Forward | provided by Mir-X^TM^ miRNA First-Strand Synthesis Kit (Code No 638315, TaKaRa) |
| U6-Reverse | provided by Mir-X^TM^ miRNA First-Strand Synthesis Kit (Code No 638315, TaKaRa) |

**Supplementary Table 2.** Gene primers for qRT-PCR.

| **Gene Name** | **Primer Sequence (5’ to 3’)** |
| --- | --- |
| ATP2B4-forward | AGATGTCACGCTTATCATCCTGG |
| ATP2B4-reverse | TTGCGACTTGACCACACAGT |
| GAPDH-forward | ACAACTTTGGTATCGTGGAAGG |
| GAPDH-reverse | GCCATCACGCCACAGTTTC |

**Supplementary Table 3.** Screening miRNA data sets by Venn diagram.

| **List Names** | **Chip or Data Sets** | **Number of Unique Elements** |
| --- | --- | --- |
| MicroRNA Expressed by Myeloma Cells | GSE125364 | 2006 |
| MicroRNA Expressed of RBC | GSE11060 GSE98830 GSE114990 GSE32035 GSE63703 GSE65706 | 1884 |
| MicroRNA Unexpressed of RBC* | GSE63703 GSE65706 | 926 |
| TargetScanHuman 7.2 | Target “ATP2B4” | 1198 |
| miRDB | Target “ATP2B4” | 297 |
| Overall number of unique elements | - | 3017 |

- * The number of chip copies read is 0.

**Supplementary Table 4.** Evaluation criteria of RBCs’ oxidative damage model.

| **Classification** |  | **Score** | **Evaluation Criterion of RBCs** |
| --- | --- | --- | --- |
| − |  | 0 | Normal morphology |
| + |  | 1 | Single punctate corpuscles appear in RBCs |
| ++ |  | 2 | Number of punctate bodies in RBCs ≥ 2 or form linear corpuscles < 2/3 of the circumference of RBCs |
| +++ |  | 3 | Linear corpuscles ≥ 2/3 of the circumference of RBCs or ≥ 2 linear corpuscles or large corpuscles can be seen in RBCs |

Notes：Count 500 cells, divide the change of RBCs morphology into four levels, calculate the percentage of various types of RBCs, and then multiply the percentage with the corresponding integral respectively. The total integral obtained is used for later evaluation. [(“−”%) + (“+”%) + (“++”%) + (“+++”%) =100%]. The total integral = [(“−”%)×0’] + [(“+”%)×1’] + [(“++”%)×2’] + [(“+++”%)×3’].

**Supplementary Table 5.** Effect of omega-Agatoxin IVA on intracellular Ca^2+^ content in RBCs (Concentration of T-BHP: 100nM).

|  | **Concentration of T-BHP: 100nM** | | | |
| --- | --- | --- | --- | --- |
| **Concentration of omega-Agatoxin IVA** | **Time of RBCs treated with T-BHP** | **mean value** | **Standard error** | **P value** |
| **0nM** | 0min | 1.059449588 | 0.020404737 | / |
|  | 1min | 2.048679723 | 0.021040755 | / |
|  | 2min | 2.149945014 | 0.009856585 | / |
|  | 4min | 1.939128576 | 0.01379328 | / |
|  | 8min | 0.674710491 | 0.038986483 | / |
|  | 10min | 0.613229129 | 0.003371183 | / |
| **0.5nM** | 0min | 1.098778159 | 0.010091135 | / |
|  | 1min | 2.430377509 | 0.024228845 | 0.00497 |
|  | 2min | 2.364239652 | 0.006821808 | 0.00109 |
|  | 4min | 2.196893906 | 0.006339174 | 0.00132 |
|  | 8min | 0.647473142 | 0.00959425 | / |
|  | 10min | 0.624424981 | 0.005480046 | / |
| **1nM** | 0min | 1.114496069 | 0.016823018 | / |
|  | 1min | 2.597431976 | 0.010076413 | 3.76E-04 |
|  | 2min | 2.391952459 | 0.006628449 | 6.57E-04 |
|  | 4min | 2.372189235 | 0.006329688 | 1.77E-04 |
|  | 8min | 0.782039911 | 0.014040666 | / |
|  | 10min | 0.572910656 | 0.018499594 | / |
| **2nM** | 0min | 1.156632976 | 0.026243047 | / |
|  | 1min | 2.082254995 | 0.00697224 | / |
|  | 2min | 1.938254433 | 0.072660007 | / |
|  | 4min | 1.891738072 | 0.043243032 | / |
|  | 8min | 0.884777018 | 0.037949746 | / |
|  | 10min | 0.61456248 | 0.015628999 | / |
| **4nM** | 0min | 0.938455909 | 0.009960958 | / |
|  | 1min | 1.255622561 | 0.003504463 | 6.22E-05 |
|  | 2min | 1.4571295 | 0.043637192 | 0.00187 |
|  | 4min | 1.507324554 | 0.007401737 | 2.02E-04 |
|  | 8min | 0.520290479 | 0.005584836 | / |
|  | 10min | 0.500986517 | 0.011401186 | / |
| **8nM** | 0min | 1.03034362 | 0.013554225 | / |
|  | 1min | 1.21673744 | 0.02107692 | 1.92E-04 |
|  | 2min | 1.578721362 | 0.08636665 | 0.0363 |
|  | 4min | 1.317877515 | 0.047282351 | 0.00401 |
|  | 8min | 0.644520233 | 0.014245584 | / |
|  | 10min | 0.568697391 | 0.010624718 | / |

Notes：*P* value, Compared with the omega-Agatoxin IVA concentration of 0 nM group. nM, nmol·L^-1^.

**Supplementary Table 6.** Effect of omega-Agatoxin IVA on intracellular Ca^2+^ content in RBCs (Concentration of T-BHP: 250nM).

|  | **Concentration of T-BHP: 250nM** | | | |
| --- | --- | --- | --- | --- |
| **Concentration of omega-Agatoxin IVA** | **Time of RBCs treated with T-BHP** | **mean value** | **Standard error** | **P value** |
| **0nM** | 0min | 1.188180475 | 0.005523708 | / |
|  | 1min | 1.548657188 | 0.001225265 | / |
|  | 2min | 2.480456862 | 0.016215254 | / |
|  | 4min | 1.222882579 | 0.013420423 | / |
|  | 8min | 0.396627083 | 0.002096105 | / |
|  | 10min | 0.763035083 | 0.002673236 | / |
| **0.5nM** | 0min | 1.232844962 | 0.003228715 | / |
|  | 1min | 1.650275828 | 0.052673335 | / |
|  | 2min | 2.711092129 | 0.002813083 | 0.00272 |
|  | 4min | 1.051691938 | 0.010188059 | 0.00871 |
|  | 8min | 0.744874919 | 0.016603855 | 6.05E-04 |
|  | 10min | 0.688283103 | 0.013450325 | / |
| **1nM** | 0min | 1.214213751 | 0.006238305 | / |
|  | 1min | 2.407945968 | 0.01581503 | 1.40E-05 |
|  | 2min | 3.262885782 | 0.015103264 | 7.63E-05 |
|  | 4min | 0.969668383 | 0.000766956 | 8.89E-04 |
|  | 8min | 0.524041363 | 0.013680675 | 1.23E-02 |
|  | 10min | 0.3518497 | 0.007539252 | 1.72E-05 |
| **2nM** | 0min | 1.200161618 | 0.007649375 | / |
|  | 1min | 1.415338781 | 0.013281077 | 0.00923 |
|  | 2min | 1.875309891 | 0.006769031 | 8.42E-05 |
|  | 4min | 0.841112022 | 0.041307231 | 0.01433 |
|  | 8min | 0.63015888 | 0.009454589 | 3.41E-04 |
|  | 10min | 0.337625441 | 0.004359396 | 2.53E-06 |
| **4nM** | 0min | 1.214607078 | 0.006826428 | / |
|  | 1min | 1.469685891 | 0.074314888 | / |
|  | 2min | 1.664314964 | 0.074940613 | 0.0074 |
|  | 4min | 1.032283115 | 0.079891562 | / |
|  | 8min | 0.347204862 | 0.002408713 | 0.00188 |
|  | 10min | 0.956565928 | 0.035462949 | / |
| **8nM** | 0min | 1.221112484 | 0.006938473 | / |
|  | 1min | 1.821875616 | 0.014473109 | 8.93E-04 |
|  | 2min | 1.863836934 | 0.019743511 | 3.40E-04 |
|  | 4min | 1.022259638 | 0.041519447 | / |
|  | 8min | 0.423239316 | 0.002298302 | 0.01569 |
|  | 10min | 0.833706159 | 0.059736846 | / |

Notes：*P* value, Compared with the omega-Agatoxin IVA concentration of 0 nm group. nM, nmol·L^-1^.

**Supplementary Table 7.** Effect of omega-Agatoxin IVA on intracellular ROS content in RBCs (Concentration of T-BHP: 100nM).

|  | **Concentration of T-BHP: 100nM** | | | |
| --- | --- | --- | --- | --- |
| **Concentration of omega-Agatoxin IVA** | **Time of RBCs treated with T-BHP** | **mean value** | **Standard error** | **P value** |
| **0nM** | 0min | 20.32271229 | 0.219143173 | / |
|  | 1min | 24.40022926 | 0.279433134 | / |
|  | 2min | 30.11417518 | 0.565690909 | / |
|  | 4min | 34.27087002 | 1.324894224 | / |
|  | 8min | 184.9921824 | 2.622023253 | / |
|  | 10min | 379.3685753 | 6.985116384 | / |
| **0.5nM** | 0min | 19.90369747 | 0.139652235 | / |
|  | 1min | 29.80523326 | 0.586200147 | 0.0172 |
|  | 2min | 34.46686172 | 0.739540808 | / |
|  | 4min | 39.44022186 | 0.920761899 | / |
|  | 8min | 211.0368981 | 1.729926186 | 1.74E-02 |
|  | 10min | 403.5224156 | 5.308147465 | / |
| **1nM** | 0min | 20.22061077 | 0.234740164 | / |
|  | 1min | 33.5485747 | 0.868178134 | 9.11E-03 |
|  | 2min | 46.82113047 | 3.32413316 | / |
|  | 4min | 56.38929996 | 1.129700799 | 3.91E-03 |
|  | 8min | 226.4056961 | 3.660492677 | 1.23E-02 |
|  | 10min | 200.1280262 | 3.704670477 | 4.34E-04 |
| **2nM** | 0min | 19.87897694 | 0.416272472 | / |
|  | 1min | 35.73201618 | 1.051337884 | 0.00798 |
|  | 2min | 46.10733182 | 2.55394468 | 4.49E-02 |
|  | 4min | 75.3594172 | 1.322207327 | 0.00049221 |
|  | 8min | 256.3560554 | 3.559137772 | 1.60E-03 |
|  | 10min | 253.3835583 | 4.682549902 | 2.12E-03 |
| **4nM** | 0min | 19.99647035 | 0.416972502 | / |
|  | 1min | 31.88234239 | 0.722105812 | 0.01038 |
|  | 2min | 34.51057928 | 0.741723004 | / |
|  | 4min | 41.52712452 | 0.927592637 | / |
|  | 8min | 221.5686414 | 3.985844639 | 0.02248 |
|  | 10min | 317.7486874 | 3.045400779 | 0.01891 |
| **8nM** | 0min | 19.76137374 | 0.133858126 | / |
|  | 1min | 34.78482684 | 0.729099993 | 3.30E-03 |
|  | 2min | 38.16580167 | 0.304060328 | 4.10E-03 |
|  | 4min | 49.90712783 | 0.461646688 | 0.00628 |
|  | 8min | 228.5516967 | 3.840391968 | 0.01155 |
|  | 10min | 263.6102657 | 3.237123919 | 0.00209 |

Notes：*P* value, Compared with the omega-Agatoxin IVA concentration of 0 nm group. nM, nmol·L^-1^.

**Supplementary Table 8.** Effect of omega-Agatoxin IVA on intracellular ROS content in RBCs (Concentration of T-BHP: 250nM).

|  | **Concentration of T-BHP: 250nM** | | | |
| --- | --- | --- | --- | --- |
| **Concentration of omega-Agatoxin IVA** | **Time of RBCs treated with T-BHP** | **mean value** | **Standard error** | **P value** |
| **0nM** | 0min | 19.6785811 | 0.424917795 | / |
|  | 1min | 26.3873449 | 0.453237119 | / |
|  | 2min | 30.21686103 | 0.458466935 | / |
|  | 4min | 34.4823771 | 0.661587765 | / |
|  | 8min | 37.74220333 | 0.820033483 | / |
|  | 10min | 63.88340811 | 1.536229759 | / |
| **0.5nM** | 0min | 19.54582002 | 0.485881621 | / |
|  | 1min | 28.99521909 | 0.610772981 | / |
|  | 2min | 33.3060216 | 0.30278613 | / |
|  | 4min | 37.40821463 | 0.502654436 | / |
|  | 8min | 34.06707688 | 0.759994346 | / |
|  | 10min | 45.59627441 | 1.010909598 | 0.00939 |
| **1nM** | 0min | 20.34613958 | 0.235675009 | / |
|  | 1min | 31.94684927 | 0.581988798 | 2.37E-02 |
|  | 2min | 35.51059146 | 0.461187423 | 0.0185 |
|  | 4min | 42.92016967 | 0.781907643 | 1.78E-02 |
|  | 8min | 36.54105968 | 0.642852527 | / |
|  | 10min | 41.18580829 | 0.764669442 | 3.37E-03 |
| **2nM** | 0min | 21.66687955 | 0.072742999 | / |
|  | 1min | 30.19658702 | 0.648978051 | / |
|  | 2min | 30.88953686 | 0.776318991 | / |
|  | 4min | 32.44531906 | 0.582478967 | / |
|  | 8min | 31.22104377 | 0.669165067 | 4.39E-02 |
|  | 10min | 40.85523086 | 0.723894155 | 3.07E-03 |
| **4nM** | 0min | 20.92914397 | 0.239006088 | / |
|  | 1min | 31.70759829 | 0.603236887 | 0.02927 |
|  | 2min | 29.10770334 | 0.567697903 | / |
|  | 4min | 32.13814519 | 0.720826467 | / |
|  | 8min | 36.62764197 | 0.864407132 | / |
|  | 10min | 38.54263093 | 0.774698777 | 0.00226 |
| **8nM** | 0min | 20.48835797 | 0.075940351 | / |
|  | 1min | 23.63129909 | 0.43091266 | / |
|  | 2min | 26.63616425 | 0.229384108 | 3.01E-02 |
|  | 4min | 42.56310753 | 0.495468435 | 0.00997 |
|  | 8min | 44.44757606 | 0.638180471 | 0.03833 |
|  | 10min | 47.72381369 | 0.734047712 | 0.01104 |

Notes：*P* value, Compared with the omega-Agatoxin IVA concentration of 0 nm group. nM, nmol·L^-1^.
